# Short‐term rapamycin administration elevated testosterone levels and exacerbated reproductive disorder in dehydroepiandrosterone‐induced polycystic ovary syndrome mice

**DOI:** 10.1186/s13048-021-00813-0

**Published:** 2021-05-04

**Authors:** Zaixin Guo, Xiaohan Chen, Penghui Feng, Qi Yu

**Affiliations:** Department of Obstetrics and Gynecology, Peking Union Medical College Hospital, Peking Union Medical College and Chinese Academy of Medical Sciences, Beijing, China

**Keywords:** Polycystic ovary syndrome, mTOR, Rapamycin, Steroidogenesis, Hyperandrogenism

## Abstract

**Background:**

Polycystic ovary syndrome (PCOS) is a multifactorial endocrinopathy that affects reproduction and metabolism. Mammalian target of rapamycin (mTOR) has been shown to participate in female reproduction under physiological and pathological conditions. This study aimed to investigate the role of mTOR complex 1 (mTORC1) signaling in dehydroepiandrosterone (DHEA)-induced PCOS mice.

**Results:**

Female C57BL/6J mice were randomly assigned into three groups: control group, DHEA group, and DHEA + rapamycin group. All DHEA-treated mice were administered 6 mg/100 g DHEA for 21 consecutive days, and the DHEA + rapamycin group was intraperitoneally injected with 4 mg/kg rapamycin every other day for the last 14 days of the DHEA treatment. There was no obvious change in the expression of mTORC1 signaling in the ovaries of the control and DHEA groups. Rapamycin did not protect against DHEA-induced acyclicity and PCO morphology, but impeded follicle development and elevated serum testosterone levels in DHEA-induced mice, which was related with suppressed *Hsd3b1*, *Cyp17a1*, and *Cyp19a1* expression. Moreover, rapamycin also exacerbated insulin resistance but relieved lipid metabolic disturbance in the short term.

**Conclusions:**

Rapamycin exacerbated reproductive imbalance in DHEA-induced PCOS mice, which characterized by elevated testosterone levels and suppressed steroid synthesis. This underscores the need for new mTORC1-specific and tissue-specific mTOR-related drugs for reproductive disorders.

## Background

Polycystic ovary syndrome (PCOS) is a multifactorial disorder affecting 6–20 % of premenopausal women [1, 2]. According to the Rotterdam consensus criteria, the PCOS diagnosis requires two of the following three features: excess androgen, oligo-ovulation, or polycystic ovarian morphology [3]. PCOS is also associated with insulin resistance, type 2 diabetes, obesity, dyslipidemia, and cardiovascular disease [4]. Despite the harmful effects of PCOS on women’s health, little is known about its etiology.

The mammalian target of rapamycin (mTOR) is a conserved serine/threonine kinase of the phosphatidylinositol kinase-related kinase family that regulates cell growth, metabolism, and autophagy [5]. mTOR contains two distinct multiprotein complexes known as mTOR complex 1 (mTORC1) and mTOR complex 2 (mTORC2), depending on the sensitivity of each molecule to rapamycin [6]. mTORC1 signaling is recognized as an important factor in regulating female reproductive functions, and is indispensable in folliculogenesis, oocyte meiotic maturation, and steroidogenesis [7–10]. In addition, mTORC1 signaling is regarded as a critical regulator of glucose metabolism and lipogenesis, which is hyperactivated in obesity and type 2 diabetes [11]. Inhibition of mTORC1 can protect against diet-induced obesity and enhance insulin sensitivity [11].

Based on these observations, we hypothesize that mTORC1 signaling is involved in both reproductive and metabolic dysfunctions associated with PCOS. Several studies have reported altered expression of mTOR signaling in the ovaries of PCOS mice models and in luteal granulosa cells of patients with PCOS upon stimulation with insulin, with inconsistent conclusions [12, 13]. In this study, the change in mTORC1 signaling in the ovaries of PCOS mice was further confirmed. Rapamycin, an mTORC1 inhibitor, was transiently used in a mouse model of PCOS in order to understand the related alterations in ovarian activity and metabolic changes.

## Materials and methods

### Animal experiments

Immature (21-day-old) female C57BL/6J mice, supplied by the Vital River Laboratories of Beijing, were subcutaneously injected daily with 6 mg/100 g body weight (BW) dehydroepiandrosterone (DHEA; Sigma-Aldrich, St. Louis, MO, USA) per 0.05 mL of sesame oil for 21 consecutive days to induce hyperandrogenic PCOS. Control animals were injected with 0.05 mL of sesame oil. Subsequently, DHEA-treated mice were assigned into two experimental groups. One group was intraperitoneally injected every other day with 4 mg/kg BW rapamycin reconstituted in dimethyl sulfoxide at 125 mg/mL (Selleck Chemicals, Houston, TX, USA) diluted in phosphate-buffered saline (PBS) for the last 14 days of the DHEA treatment. The other group was treated with the vehicle for the same duration. Mice were maintained on a 12-h light/12-h darkness cycle and were provided with food and water ad libitum. Mice were group-housed with up to six mice per cage. All procedures were approved by the Committee on the Ethics of Animal Experiments of Peking Union Medical College Hospital.

### Assessment of the estrous cycle

Vaginal smears were collected on glass slides in 30 µL of PBS and then observed directly under a light microscope with a 10× objective [14]. The four stages of the estrous cycle were determined as previously described by analyzing the proportion of three major cell types (epithelial cells, cornified cells, and leukocytes) [15]. Consistent cycles of proestrus, estrus, metestrus, and diestrus (4–5 days total) in mice were called “regular cycles.”

### Ovary preparation, histology, and immunohistochemistry (IHC) analysis

Dissected ovaries were weighed, fixed in 4 % paraformaldehyde overnight at 4 °C and stored in 70 % ethanol before histological processing. For histological examination of ovarian morphology, ovaries were embedded in paraffin, serially sectioned into 5-µm thick slices, and stained with hematoxylin and eosin (H&E). Ovarian sections prepared as described above were stained to demonstrate the effects of treatment. Paraffin-embedded ovarian sections were warmed and serially deparaffinized in xylene and ethanol, introduced into an antigen unmasking solution and blocked, and incubated overnight at 4 °C with 1:400-diluted antibodies against Ser235/236 phospho-S6 kinase (catalog no. 2211) from Cell Signaling Technology (Danvers, MA, USA).

### White adipose tissue analysis

One ovarian fat pad from each mouse was fixed in 4 % paraformaldehyde overnight at 4 °C, embedded in paraffin, and sectioned into 8 μm slice. Sections were stained with H&E before images were obtained for histomorphometry at 20× magnification under a light microscope. Two representative micrographs were obtained per sample, with at least 200 μm separation between these sections.

Adipocyte size was quantified using ImageJ software (Fiji). In brief, micrographs were transformed to 8-bit grayscale, and the threshold was set to cover the adipocyte (= lipid droplet) areas and exclude anomalies such as blood vessels. Thereafter, the micrographs were transformed to a black-and-white binary image in which broken adipocyte plasma membranes were mended by applying the watershed function. The adipocytes were further defined by circularity (0.5–1.0), on which the cell area was determined.

### Insulin and glucose tolerance tests

Insulin tolerance tests (ITTs) and glucose tolerance tests (GTTs) were carried out as previously reported, with modifications [16]. Six-hour-fasted mice underwent a GTT by receiving an i.p. injection with 2 g of glucose per kg of BW. Blood glucose was measured at 0, 15, 30, 60, 90, and 120 min. For ITT, three-hour-fasted mice were intraperitoneally injected with 1 IU porcine insulin per kg of BW. Blood glucose levels were measured at 0, 15, 30, 45, and 60 min for ITTs. Blood was obtained from a tail prick, and blood glucose was measured on glucose strips and an Accu-Chek glucometer (Roche, Diagnostics, Indianapolis, IN, USA). ITTs and GTTs were carried out on day 18 of treatment.

### Serum analysis

To avoid the influence of different estrous cycle stages on the levels of serum hormones, orbital blood was obtained at diestrus in control mice under anesthetized conditions with pentobarbital (3 mg/100 mL, i.p.) after fasting for six hours, while samples were taken from the DHEA-exposed mice regardless of estrus phase because these mice were acyclic. Serum (350 µL) was separated by centrifugation at 4 °C and stored at − 80 °C until analysis. Serum luteinizing hormone (LH) and follicle-stimulating hormone (FSH) levels were assayed using a mouse ELISA kit (JM-02865M1 and JM-02838M1; Jiangsu Jingmei Biological Technology Co. Ltd, China). Serum testosterone levels were analyzed using a testosterone ELISA kit (IB79174; IBL-America, Minneapolis, MN, USA). Levels of triglycerides (TG), total cholesterol (T-CHO), low-density lipoprotein cholesterol (LDL-C), and high-density lipoprotein cholesterol (HDL-C) were measured using ELISA kits provided by Jiangsu Jingmei Biological Technology Co. Ltd (Jiangsu, China).

### Pancreatic insulin content

For the insulin content assay, the isolated pancreas was frozen in liquid nitrogen and stored at − 80 °C until use. The pancreas was thawed and homogenized with an acid/ethanol solution (0.18 M HCl, 70 % ethanol). The homogenates were stored overnight at 4 °C and then centrifuged (10,000 × g; 20 min; 4 °C), and the supernatant was prepared for the insulin assay (10-1247-01, Mercodia Inc, Sweden). The total protein concentration was measured using a protein assay kit from Beyotime Biotechnology (Shanghai, China). Pancreatic insulin content was normalized to total protein content in each sample.

### Western blotting

Ovarian proteins were extracted using RIPA lysis buffer (Beyotime Biotechnology, Shanghai, China) with protease inhibitor cocktails (No. 04693132001, Roche, USA) and phosphatase inhibitor cocktail (No. 04906845001, Roche, USA). They were separated by electrophoresis and transferred to polyvinylidene fluoride membranes. After blocking in 1% bovine serum albumin-TBST (tris-buffered saline containing 0.1 % Tween 20) for 60 min, the membranes were incubated overnight at 4 °C with specific antibodies. Antibodies against rpS6 (catalog no. 2217), p-rpS6 (S235/236) (catalog no. 2211), and β-actin (catalog no. 4970) were purchased from Cell Signaling Technology. Horseradish peroxidase-conjugated goat anti-rabbit IgG (Zhong Shan Jin Qiao, Beijing, China) were used to detect proteins through enhanced chemiluminescence and autoradiography using x-ray film.

### RNA preparation and real‐time PCR

Total RNA was isolated from 7 to 8 ovaries in each group using an RNAprep pure Tissue Kit (TIANGEN Biotech, Beijing, China) according to the manufacturer’s instructions and was quantified with a spectrophotometer (NanoDrop 2000c; Thermo Fisher Scientific, Waltham, MA, USA). RNA (500 ng/reaction per sample) was reverse-transcribed using a FastQuant RT Kit (TIANGEN Biotech) to obtain cDNA. Quantitative real-time PCR was performed using Eva Green qPCR Master Mix (Applied Biological Materials Inc., Richmond, BC, Canada) on an ABI Step One Plus platform (Thermo Fisher Scientific). The specificity of the PCR products was assessed by melting curve analyses, and amplicon size was determined by electrophoresis in 2 % agarose gels. Quantification of various mRNAs was performed using the Gapdh amplification signal as an internal control. The specific primers used are shown in Table 1.

**Table 1:** Sequences of primers used in qRT-PCR

	Forward primer	Reverse primer
*Cyp11a1*	GTACCCTGGTGTCCTTTATAGCCTCCT	TGTGTGCCATCTCATAAAGGTTCCACT
*Cyp17a1*	GCTGGCCAGAGAAGTGCTCGT	CTTGGTCCGACAAGAGGCCTAGAG
*Hsd3b1*	CTGTTGTCATCCACACTGCTGCTG	TGCTTGAACACAGGCCTCCAATAG
*Cyp19a1*	CAGCCCCTGACACCATGTCG	ACAGGCTGGTACCGCATGCTT
*Fshr*	TTTTCCAGGGAGCCTCTGGGC	AAGCCATGGTTGGGCAGGGAA
*Lhr*	CCTCCAGAGAAAAATTCACCAGCCTACT	TCGCTTCTCTAACTGTGCTTTCACATTG
*Gapdh*	AATGGATTTGGACGCATTGGT	TTTGCACTGGTACGTGTTGAT

### Statistical analysis

Data are shown as the mean ± SEM. Comparisons between groups were made using one-way ANOVA with Tukey’s post hoc test. Differences between groups with P < 0.05 were considered significant. All analyses were performed using GraphPad Prism software (GraphPad Software Inc., San Diego, CA, USA).

## Results

### Rapamycin did not protect DHEA-induced acyclicity and PCO morphology

**Fig. 1:**
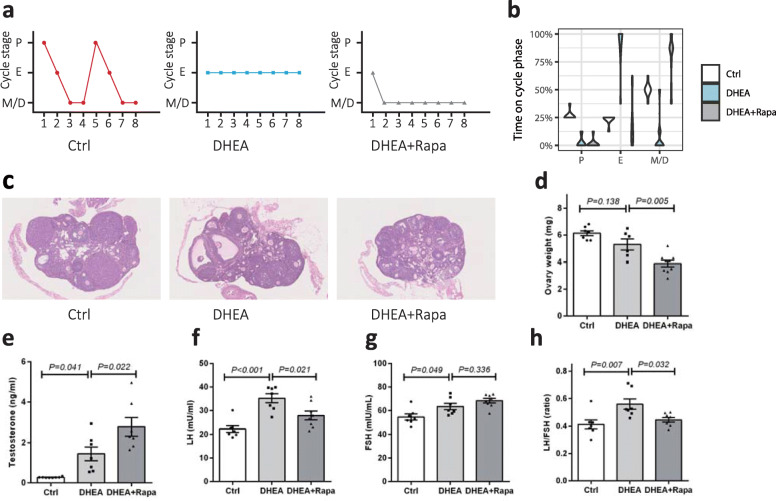
Phenotypic evaluation of control; dehydroepiandrosterone (DHEA), and DHEA + rapamycin mice. **a** Representative estrous cyclicity assessment in 5-week-old female mice for eight consecutive days by vaginal cytology. The graph shows representative cycles from the three groups. Each box indicates one animal, and dots represent a day. M, metestrus; D, diestrus; P, proestrus; E, estrus. **b** Data are presented in a violin plot showing the frequency distribution curves. **c** Ovarian morphology by H&E staining. **d** Mean ovarian weights. Analysis of serum concentrations of testosterone (**e**), luteinizing hormone (LH) (**f**), follicle-stimulating hormone (FSH) (**g**), and LH/FSH (**h**) as measured by ELISA (*n* = 7–8/group). The data are presented as means ± SEM

To investigate the effects of rapamycin administration on the DHEA-induced PCOS, we monitored estrous cyclicity, follicle population, and sex hormone levels. As expected, mice in the control group showed normal estrous cyclicity, while DHEA treatment resulted in continuous anestrus (mostly pseudoestrus). Rapamycin administration did not prevent loss of estrous cyclicity induced by DHEA, which was spent in the pseudodiestrus cycle most of the time (Fig. 1a and b). Consistent estrous cyclicity results, all ovaries of the control group contained fresh corpora lutea, which indicated recent ovulation, whereas DHEA-exposed mice, irrespective of rapamycin treatment, showed little or no corpora lutea in the ovaries (Fig. 1c). In addition, the ovaries in DHEA-treated mice presented PCO morphology, including follicular cysts, increased theca cell layer, and decreased granulosa cell layer. Ovarian weight was not affected by DHEA but decreased after the administration of rapamycin (Fig. 1d).

Undetectable levels of serum testosterone were found in control mice under evaluation. In contrast, DHEA exposure resulted in elevated serum testosterone levels. Rapamycin further significantly increased testosterone levels (*P* = 0.022; Fig. 1e) compared to DHEA treatment. FSH levels were similar among all groups (Fig. 1g), but LH levels and the LH/FSH ratio increased in response to DHEA exposure. Rapamycin decreased LH levels and the LH/FSH ratio (Fig. 1f and h).

### Treatment with DHEA did not significantly alter mTOR signaling in ovaries

**Fig. 2:**
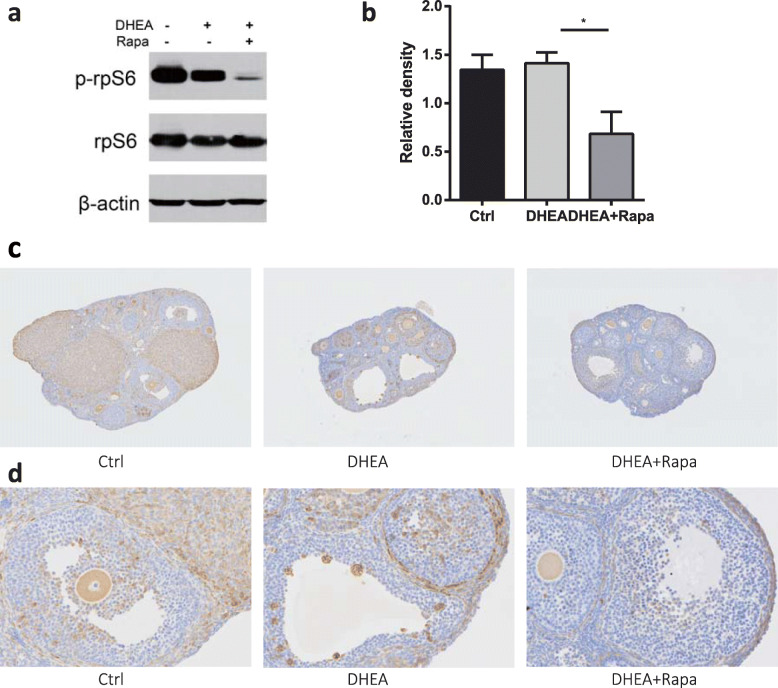
Rapamycin downregulates downstream expression of p-rpS6 pathway in the ovary. **a** Western blots of ovarian proteins with specific antibodies for p-rpS6 (S235/236), rpS6, and β-actin. Densitometry of western blots was quantified and is shown by p-rpS6 (S235/236)-to-rpS6 ratios (*n* = 6/group). The data are presented as means ± SEM. **b** Representative results from p-rpS6 immunohistochemistry are shown. (**d**) is higher magnifications of insets in (**c**)

We further performed a western blot analysis to measure the expression of phosphorylated rpS6, the mTORC1 downstream target, in the ovaries; p-rpS6 did not change as much in DHEA-induced ovaries. However, rapamycin significantly reduced p-rpS6 expression in ovaries exposed to DHEA (*P*=0.023, Fig. 2a and b).

Following immunoblotting of whole-ovary lysates, IHC was used to assess the activity of mTORC1 using rpS6 phosphorylation. P-rpS6 was strongly expressed in oocytes, cumulus cells, peri-antral granulosa cells, theca cells, and luteal cells in control and DHEA ovaries (Fig. 2c and d). Phosphorylation of rpS6 was effectively blocked by rapamycin (Fig. 2c and d).  

### Rapamycin decreased genes involved in sex hormone steroidogenesis in ovaries

**Fig. 3:**
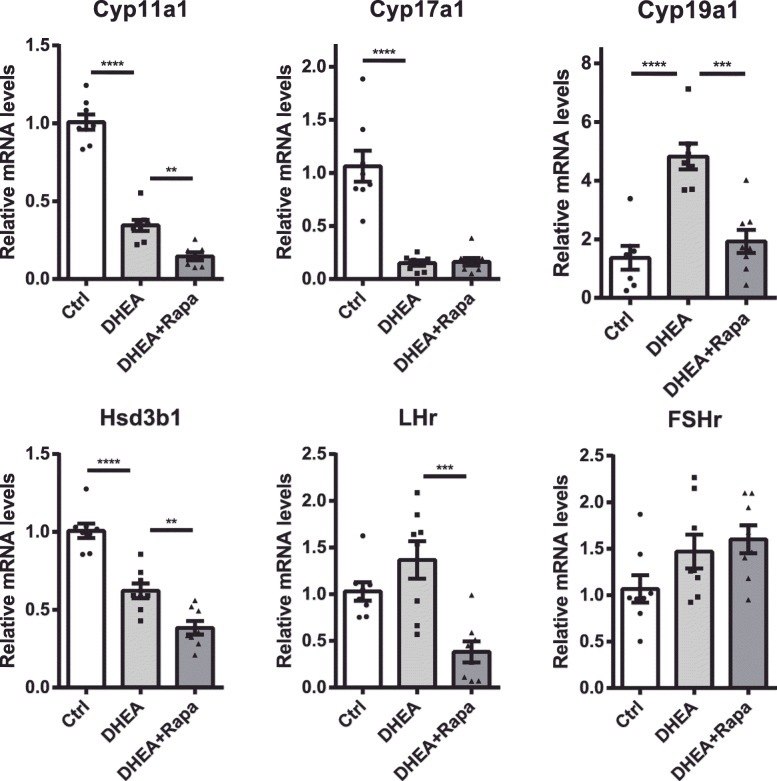
Mean mRNA expression levels of *Cyp11a1*, *Cyp17a1*, *Cyp19a1*, *Hsd3b*, *Fshr*, and *Lhr* in the ovaries from control, DHEA, and DHEA + rapamycin mice (n = 7–8/group). The data are presented as means ± SEM. *P < 0.05, **P < 0.01, *** *P* < 0.001, **** *P* < 0.0001

Using qPCR, we measured the ovarian gene expression of four enzymes involved in sex hormone steroidogenesis: 3β-hydroxysteroid dehydrogenase, 11α-hydroxylase, 17α-hydroxylase, and 19α-hydroxylase encoded by *Hsd3b1, Cyp11a1, Cyp17a1*, and *Cyp19a1*, respectively. DHEA exposure significantly affected all these enzymes. Apart from *Cyp19a1*, the expression levels of all enzymes decreased. Rapamycin decreased *Hsd3b1*, *Cyp17a1*, and *Cyp19a1* expression. Moreover, rapamycin-induced testosterone expression was increased probably due to the conversion of androgens to estrogens catalyzed by *Cyp19a1*. In addition, the expression of *Lhr*, a receptor for LH and human chorionic gonadotropin (hCG) involved in ovulation, dramatically decreased after treatment with rapamycin (Fig. 3).

### Rapamycin worsened glucose metabolism

**Fig. 4:**
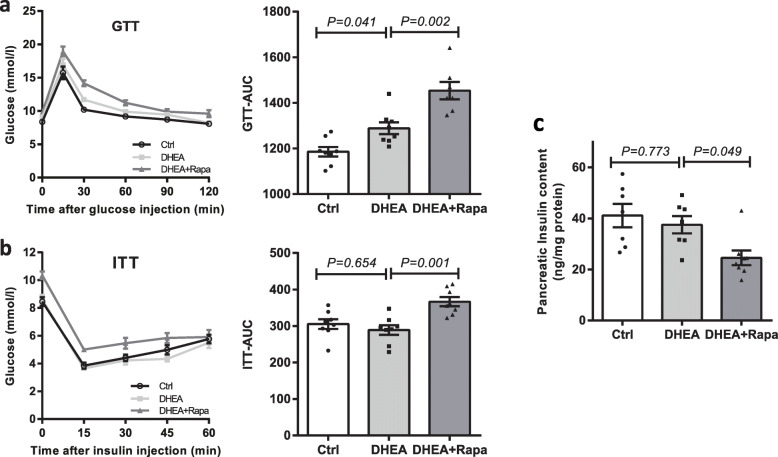
Oral glucose tolerance test (GTT; **a**), insulin tolerance test (ITT; **b**), and pancreatic insulin content (**c**). *n* = 7–8/group in **a**-**c**. The data are presented as means ± SEM

Glucose tolerance was lower in DHEA-exposed mice than in controls as determined by the area under the curve during GTTs. DHEA-exposed mice had similar insulin sensitivity levels compared to controls. After treatment with rapamycin, significantly lower glucose tolerance and more insulin resistance were found compared to DHEA-exposed mice without rapamycin (Fig. 4a and b). The DHEA-treated mice tended to have decreased pancreatic insulin content, and rapamycin treatment further reduced it significantly (Fig. 4c).

### Rapamycin protected against DHEA-induced adipocyte hypertrophy and dyslipidemia

**Fig. 5:**
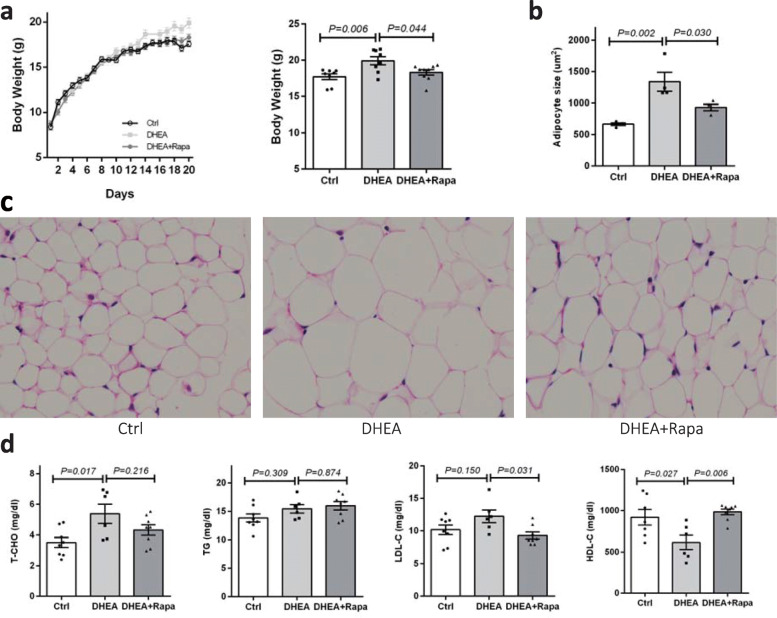
Body weight, adipocyte size of gonadal adipose tissue, and serum lipid levels in control; dehydroepiandrosterone (DHEA), and DHEA + rapamycin mice. **a** Body weight gain and body weight after treatment (*n* = 8/group); **b** Adipocyte size of gonadal adipose tissue (*n* = 4/group); **c** Histological sections showing adipose tissue; **d** Serum lipid levels (*n* = 6–8/group). The data are presented as means ± SEM

BW gain was significantly greater in DHEA-treated mice than in control mice. Rapamycin treatment significantly decreased BW (*P* = 0.044; Fig. 5a). The mean adipocyte size in gonadal adipose tissue was about two-fold larger in DHEA-exposed mice than in controls. Rapamycin treatment significantly led to smaller adipocytes in DHEA-induced mice and prevented DHEA-induced adipocyte hypertrophy (Fig. 5b and c). Furthermore, serum levels of T-CHO levels were significantly higher in DHEA-exposed mice which were reduced by rapamycin. Rapamycin also decreased DHEA-induced elevated LDL-C and increased DHEA-induced suppression of HDL-C. However, serum TG levels were elevated by DHEA, although not significantly, and were rarely affected by rapamycin (Fig. 5d).

## Discussion

The mTOR pathway is regarded as an important pathophysiological basis for PCOS, but the effects of related drugs on a PCOS mouse model have rarely been investigated. In the current study, we showed that rapamycin worsened the development of reproductive dysfunction and increased DHEA-induced elevated testosterone, which was probably related to suppressed steroid synthesis. Rapamycin also exacerbated insulin resistance but relieved lipid metabolic disturbance in the short term.

mTOR modulates many key processes at the cellular level, including protein synthesis, biomass accumulation, and autophagy, and coordinates the storage and mobilization of nutrients and energy at the organismal level [11]. As a critical regulator of metabolic syndrome, the mTOR pathway underwrites many diseases of overeating, including obesity and type 2 diabetes. Furthermore, inhibition of the mTORC1 might reverse the metabolic dysregulation and diseases [11]. The mTOR pathway is essential for folliculogenesis and steroidogenesis in the ovary [7, 17]. Blockade of mTORC1 also decreases LH secretion in rodents, which is regulated by the neuroendocrine hypothalamus [18]. Thus, it can be assumed that the mTOR pathway participates in the development of PCOS, a common endocrinopathy disease involving both reproduction and metabolism.

However, the functional regulation of the mTOR pathway in PCOS is exponentially more complex because mTOR coordinates different physiological processes across different tissues. Furthermore, divergent results involving the expression of the mTOR pathway in PCOS were observed. mTOR phosphorylation was increased in the ovary of DHEA-treated PCOS mice, whereas S6K1 phosphorylation, downstream of mTORC1 and upstream of rpS6, was decreased [12]. We did not observe significant changes in the expression of rpS6 phosphorylation. We postulated that the results were partly because of the effect of mixing different kinds of cells from ovarian tissues, as proven by the IHC results that p-rpS6 was expressed in oocytes, cumulus cells, peri-antral granulosa cells, theca cells in follicles and luteal cells in corpus luteum [19]. As the mTOR pathway is expressed differently in follicles and corpus luteum, changes in the percentage of follicles and corpus luteum can lead to bias risks. Thus, the exact expression of a specific type of cell could be more accurate. mTOR expression in granulosa cells of PCOS patients has also been investigated. A recent study showed that granulosa cells of PCOS patients had higher mTOR expression and phosphorylation than those of non PCOS patients; this mTOR expression could be reduced by berberine, a quaternary compound extracted from plants regulating glucose metabolism [20]. Another study found that mTOR expression in luteal granulosa cells was similar in PCOS patients and healthy individuals, but it was less in luteal granulosa cells in PCOS patients than in controls upon stimulation with insulin [13]. Given the impacts of insulin involvement in both studies, it can be postulated that glucose metabolism affects the aberrant expression of mTOR in granulosa cells of PCOS patients.

As few differences in mTOR expression in the ovarian tissue were found between control and DHEA-treated mice, rapamycin was used as an inhibitor to investigate the impact of mTOCR1 on reproductive imbalance in PCOS. As expected, follicle development was impeded, as mTOR is indispensable for follicle development and maturation. With less mature follicle, granulosa cells expressing *Lhr* reduced, consistent with the findings of previous report [21]. Surprisingly, testosterone was further elevated following treatment with rapamycin in DHEA-exposed mice, despite LH being significantly suppressed. To explain this phenomenon, mRNA expression of genes involved in steroidogenesis was explored. Consistent with previous results, DHEA exposure decreased *Cyp11a1*, *Hsd3b*, and *Cyp17a1* expression, which was likely due to negative feedback of DHEA, but increased *Cyp19a1* expression, which converts excess DHEA into estrogens [22, 23]. Treatment with rapamycin further reduced *Cyp11a1*, *Cyp17a1*, and *Cyp19a1* expression, probably because cyclic adenosine monophosphate response element-binding protein, downstream of S6K1, could be significantly decreased by rapamycin and mediates changes in gene expression [9, 10]. This phenomenon was validated by *in vitro* experimental data, which showed that rapamycin reduced hCG-induced upregulation of *Cyp11a1*, *Hsd3b*, and *Cyp17a1* in mice interstitial cells and human granulosa lutein cells [9, 10, 24]. Excess androgens could not be converted into estrogens because of the decreased *Cyp19a1* expression mediated by rapamycin; thus, testosterone levels were further elevated. Apart from the direct influence of rapamycin on the ovaries, steroidogenesis can also be indirectly altered by changes in insulin resistance and metabolic actions induced by rapamycin, which can further exacerbate ovulatory disturbances [25].

PCOS presents insulin resistance and visceral adiposity, which results from excess androgen and further facilitates androgen secretion by the ovaries and adrenal glands [2]. Insulin resistance, adipocyte hypertrophy, and dyslipidemia were present in DHEA-induced mice in this study, validating this PCOS model as regards metabolic alterations [22]. mTORC1 is a critical regulator of glucose metabolism and lipogenesis across various tissues, and is hyperactivated in diseases caused by excessive nutrients and mitogens, including PCOS [11]. However, direct pharmacological inhibition of mTORC1 by rapamycin in DHEA-exposed mice exacerbated insulin resistance [11]. This is probably because rapamycin also inhibits mTORC2 in addition to mTORC1 in a dose-, time- and cell-type-dependent manner [26]. In contrast, rapamycin decreased BW and served as a negative regulator of adipocyte development in DHEA-exposed mice. Furthermore, rapamycin significantly decreased LDL-C and increased HDL-C, which relieved dyslipidemia. Despite relieving the dysregulation of lipid metabolism in DHEA-mice in the short term, rapamycin has been shown to lead to ectopic accumulation of lipids in the muscle and liver on the long term [27].

The pathogenesis and development of PCOS can be described as a vicious circle in which excess androgens lead to insulin resistance, hyperinsulinism, and abdominal visceral adiposity, further facilitating androgen secretion by the ovaries and adrenal glands [2]. Thus, drugs treating any parts of this vicious circle can theoretically be used to cure PCOS. On the other hand, drugs aggravating any part of the circle can also exacerbate the disease. The mTOR pathway plays a central role in maintaining cellular homeostasis and is active in various tissues, indicating that suppression of mTOR could affect the whole body [11]. Taking this study as an example, rapamycin suppressed the ovulation and steroid synthesis, elevating testosterone. Testosterone elevation further increased insulin resistance, and finally aggravated steroidogenesis imbalance. Thus, it is important to interpret results systematically. Furthermore, it is probably more practical to transform systemically functional drugs into tissue-specific targeted drugs to relieve their side effects.

The strength of the study is that we systematically displayed the effects of short-term rapamycin on reproduction and metabolism in DHEA-induced PCOS mice *in vivo*, which has been investigated little. We found that rapamycin further exacerbated reproductive imbalance in DHEA-induced PCOS mice by impeding follicle development, elevating testosterone, and suppressing steroid synthesis, emphasizing the necessity of evaluating these mixed functions when administering a systematic drug. Thus, mTORC1-specific and tissue-specific new drugs are needed to bypass the adverse effects resulting from direct pharmacological inhibition of mTORC1 [11]. Our study has some limitations. DHEA-induced PCOS mice cannot completely mimic the aberrant regulation of steroid metabolism in PCOS patients, who reportedly show reduced expression of CYP11A1 and HSD17B1 and increased expression of SULT1E1 in granulosa-lutein cells [28]. Thus, effects of rapamycin on steroidogenesis in PCOS patients may be different. In addition, we mainly examined rapamycin-induced reproductive imbalance in DHEA-induced PCOS mice. Hence, metabolic disturbances induced with rapamycin administration warrant further investigation to help understand PCOS pathophysiology.

## Conclusions

Our study demonstrated that serum testosterone was significantly elevated by rapamycin in DHEA-mice, which was correlated with the suppression of steroid synthesis. Furthermore, new drugs that are mTORC1-specific and tissue-specific are needed to bypass the adverse effects of rapamycin when administering mTOR-related drugs for reproductive disorders.

## Data Availability

Not applicable.

## Abbreviations

BW: Body weight
DHEA: Dehydroepiandrosterone
FSH: Follicle-stimulating hormone
GTT: Glucose tolerance test
hCG: Human chorionic gonadotropin
H&E: Hematoxylin and eosin
HDL-C: High-density lipoprotein cholesterol
IHC: Immunohistochemistry
ITT: Insulin tolerance test
LDL-C: Low-density lipoprotein cholesterol
LH: Luteinizing hormone
mTOR: Mammalian target of rapamycin
mTORC1: mTOR complex 1
mTORC2: mTOR complex 2
PBS: Phosphate-buffered saline
PCOS: Polycystic ovary syndrome
T-CHO: Total cholesterol
TG: Triglycerides
